# Spatial tactile localization depends on sensorimotor binding: preliminary evidence from virtual reality

**DOI:** 10.3389/fnhum.2024.1354633

**Published:** 2024-02-20

**Authors:** Matteo Girondini, Massimo Montanaro, Alberto Gallace

**Affiliations:** ^1^Department of Psychology, University of Milano-Bicocca, Milan, Italy; ^2^Mind and Behavior Technological Center, University of Milano-Bicocca, Milan, Italy; ^3^MySpace Lab, Department of Clinical Neuroscience, University Hospital of Lausanne, Lausanne, Switzerland

**Keywords:** tactile localization, sensorimotor binding, virtual reality, temporal order judgment (TOJ), point of subjective equality (PSE)

## Abstract

**Introduction:**

Our brain continuously maps our body in space. It has been suggested that at least two main frames of reference are used to process somatosensory stimuli presented on our own body: the anatomical frame of reference (based on the somatotopic representation of our body in the somatosensory cortex) and the spatial frame of reference (where body parts are mapped in external space). Interestingly, a mismatch between somatotopic and spatial information significantly affects the processing of bodily information, as demonstrated by the “crossing hand” effect. However, it is not clear if this impairment occurs not only when the conflict between these frames of reference is determined by a static change in the body position (e.g., by crossing the hands) but also when new associations between motor and sensory responses are artificially created (e.g., by presenting feedback stimuli on a side of the body that is not involved in the movement).

**Methods:**

In the present study, 16 participants performed a temporal order judgment task before and after a congruent or incongruent visual-tactile-motor- task in virtual reality. During the VR task, participants had to move a cube using a virtual stick. In the congruent condition, the haptic feedback during the interaction with the cube was provided on the right hand (the one used to control the stick). In the incongruent condition, the haptic feedback was provided to the contralateral hand, simulating a sort of ‘active’ crossed feedback during the interaction. Using a psychophysical approach, the point of subjective equality (or PSE, i.e., the probability of responding left or right to the first stimulus in the sequence in 50% of the cases) and the JND (accuracy) were calculated for both conditions, before and after the VR-task.

**Results:**

After the VR task, compared to the baseline condition, the PSE shifted toward the hand that received the haptic feedback during the interaction (toward the right hand for the congruent condition and toward the left hand for the incongruent condition).

**Dicussion:**

This study demonstrated the possibility of inducing spatial biases in the processing of bodily information by modulating the sensory-motor interaction between stimuli in virtual environments (while keeping constant the actual position of the body in space).

## Introduction

Every physical interaction with the external environment involving the body relies on the somatosensory system. The somatosensory system defines the physical boundaries between the body and its surroundings. Sensory receptors, widely distributed across the skin, enable the transmission of somatosensory information from the periphery to the central nervous system. However, when a physical stimulus approaches our body, somatosensory processing can occur using multiple reference systems (Heed et al., 2015). The first step in somatosensory information processing at the cortical level occurs in the primary somatosensory cortex (SI). In SI, the signal is elaborated according to the somatotopic representation of the body in the brain. In particular, the primary somatosensory cortex (SI) exhibits a topographical organization, with specific cortical regions dedicated to processing somatosensory input from different body parts (Okada et al., 1984; Ruben et al., 2001; Medina and Coslett, 2010). This mapping, as originally described by Penfield in the 1950s (Penfield and Boldrey, 1937), reflects how sensory information from various body areas is represented in the brain (Chau and Herring, 2021). However, we usually distinguish the location of the stimulation as a function of the position of our body in the surrounding space. Indeed, considering the localization of tactile input, the somatotopic reference informs the brain about the localization of the input on the skin’s surface, but it is not informative on input location in terms of external space since the body posture changes continuously (Pavani et al., 2000). For this reason, the human brain (also) maps the body and its parts in a space-based reference system (from now, spatiotopic), whereby the central body midline defines the left and right side of body-space. This spatiotopic representation (centered on the body) is based on further sensory signals, for which visual and proprioceptive information is combined with incoming somatosensory inputs, transforming somatosensory input into bodily spatial coordinates (a process known as tactile remapping). Notably, the spatiotopic and the somatotopic representation of the body are not independent; instead, they are integrated into a coherent high-level and multisensory-based body representation (Moseley et al., 2012a). In this context, the brain continuously updates and integrates information from somatotopic and spatiotopic systems to localize sensory inputs within our body’s space accurately and interact with the environment.

Nonetheless, these two reference systems (somatotopic and spatiotopic) must be aligned for optimal tactile localization. For instance, when the hands are positioned in an unconventional posture compared to the usual spatial configuration of the body, the capability of localizing somatosensory information on our body significantly decreases (Shore et al., 2002). This situation arises, for instance, when we cross hands, leading to a conflict between the somatotopic and spatiotopic representation of the body (Yamamoto and Kitazawa, 2001). Specifically, crossing the hands over the body midline deteriorates the ability to process and localize tactile stimuli presented on the hands, a phenomenon known as the “crossing-hand” effect (Sambo et al., 2013). This effect is usually investigated using a temporal order judgment task (TOJ) (Schicke and Rôder, 2006). During a TOJ task, participants have to discriminate the order of presentation of two tactile stimuli presented one to each hand with a short temporal delay between the first and the second. The participants have to indicate which hand received the first (or the second, to avoid response bias) stimulus. The delay (or SOA, e.g., stimulus onset asynchrony) between the two stimuli varies across trials, generally from 15 to 600 milliseconds. In these studies, the just notable difference, or JND, is a standard measurement used to define sensitivity in tactile localization, reflecting the delay in which participants reported correct responses in 84% of the cases (or 75%, depending on the study). Under a normal posture (uncrossed hand), participants can discriminate the order of presentation quite accurately for short delays [around ∼30 90 ms (Heed and Azañón, 2014)]. However, performance significantly decreases if the hands are crossed over the midline, for which participants often confuse the order of presentation, and a higher SOA (around ∼150–300 ms) is required to obtain accurate performance. Several studies reported decreased tactile localization when hands are crossed to the body midline (Aglioti et al., 1999; Yamamoto and Kitazawa, 2001; Schicke and Rôder, 2006; Gallace et al., 2011; Sambo et al., 2013). The main interpretation of the impairment found in tactile localization under crossed conditions refers to a conflict between the somatotopic and the spatiotopic systems, which causes a time cost in sensory processing, leading to impaired performance.

Notably, temporal order judgment has also been used to measure spatial bias toward one side of the body. This is indexed by the point of subjective equality (or PSE), which defines the temporal delay in which the two stimuli are perceived to come from the left hand in 50% of the cases and from the right hand in the rest of the trials (point of maximum uncertainty) (Wada et al., 2004; Heed and Azañón, 2014). The presence of a bias toward one side of the body was reported in some clinical conditions, such as hemispatial neglect (Berberovic et al., 2004; Van der Stigchel and Nijboer, 2018) and chronic pain regional syndrome (CRPS) (Moseley et al., 2009; Reid et al., 2018). Indeed, right brain damage often leads to spatial neglect toward the left side of the space, resulting in a decrease in performance for explicitly reporting stimuli that come from the right side of the space. Moreover, a reduction of bias for the neglected side was reported after prismatic adaptation treatment, suggesting the role of spatial attention in processing the incoming stimuli (Berberovic et al., 2004). The presence of bias in the tactile localization task was also described by Moseley and colleagues in CRPS patients (Moseley et al., 2009). CRPS is a neuropathic pain disorder characterized by chronic pain and autonomic dysfunction (altered sweating, skin temperature, and color) for a specific body part (usually involving an upper or lower limb) (Bruehl, 2010). This condition often results in a bias in processing stimuli where those coming from the unaffected side of the body are prioritized over those coming from the affected side of the body. This result can be found by measuring the PSE during a TOJ task involving affected and non-affected hands. However, crossing the hands has been shown to invert the bias, where CRPS patients prioritize the unaffected space of the body despite the presence of the affected limb, suggesting that the bias in information processing showed in CRPS patients is spatiotopic (space-based) rather than somatotopic defined (Moseley et al., 2009, 2012b).

Taken together, temporal order judgment is a useful paradigm for investigating processing related to tactile localization and spatial remapping into a unified, coherent, multisensory representation of the body. More specifically, somatosensory processing depends on both somatotopic and spatiotopic frames to localize tactile inputs presented on the body. The crossed posture paradigm, in particular, offers compelling evidence of the existence and interplay of these two reference frames. Within this framework, TOJ outcomes provide valuable information regarding spatial bias or sensitivity in localizing somatosensory information presented on the body surface. However, evidence of a mismatch between the somatotopic and the spatiotopic system has been provided only by manipulating the posture of hands (crossed vs. uncrossed). To date, no previous study has explored the effect of crossing the somatosensory feedback instead of crossing the hands, maintaining an intact spatial (left-right) representation of the body. Moreover, the crossed-hand paradigm can be considered a passive visual-proprioceptive illusion where there is no involvement of motor components (that is, no movements are performed during the task). Note, however, that our ability to localize information in space should be based on the binding between the position of the stimuli in the external environment and the feedback (visual, proprioceptive, and tactile) that we receive from the interaction with such environment. Moreover, the role of proprioceptive information under passive conditions of stimulus presentation (such as in the classical crossed-hand illusion) is certainly less relevant as compared to active motor interactions, where proprioception needs to continuously inform our brain about the current position of our body (Tuthill and Azim, 2018).

The present study aims to fill this gap and investigate the effect of crossing the tactile feedback during active movements, whereby a right-hand interaction causes a tactile stimulation of the left hand. To achieve this manipulation, we developed a novel paradigm based on Virtual Reality synchronized with a vibrotactile actuator system. Specifically, during the virtual interaction, the spatial position of the right hand remained constant and mapped into the right part of the space. However, in one of the two experimental sessions, the somatosensory feedback received from object interactions did not adhere to the spatial configuration of the corresponding body part, and it was consistently provided to the left hand, leading to a mismatch between the part of the body that performed the movement and the one that received the somatosensory feedback. That is, our employed paradigm (differently from the classic crossed hands paradigms) is an active sensorimotor task involving motor, visual-proprioceptive, and somatosensory information received during (virtual) object interaction. The primary focus of the study was to evaluate potential after-effects in somatosensory processing resulting from the artificial binding between motor commands and somatosensory feedback, received as a consequence of action wherein right-hand interaction caused left-hand tactile feedback. In a fully with-in experimental design, we measured participants’ performance in tactile localization using a temporal order judgment (TOJ) carried out at the baseline and after 10 min of the virtual reality task in both congruent and incongruent conditions.

## Materials and methods

### Participants

A total of eighteen (Female = 10) participants aged 19–63 years old (M = 27.1, SD = 13.2) were recruited by self-enrollment using the university recruiting platform. The sample size for this study was calculated using G*Power 3.1 (Faul et al., 2007) and the repeated ANOVA statistical test (four measurements and two within factors). G*Power indicated that, with α = 0.05 power (1−β) = 0.80 and a medium effect size (0.4) (Heed and Azañón, 2014), the estimated sample size for this study was at least 17 participants. Participants’ handness was assessed using Edinburgh inventory for handness (Oldfield, 1971), revealing that all participants except one were right-handed. After recruitment, two participants were excluded from the analysis due to technical problems. Sixteen participants were included in the final sample. The study was conducted according to the ethical principles of the Declaration of Helsinki and was approved by the Department of Psychology, University of Milano Bicocca, local ethical committee.

### Experimental design

**FIGURE 1:**
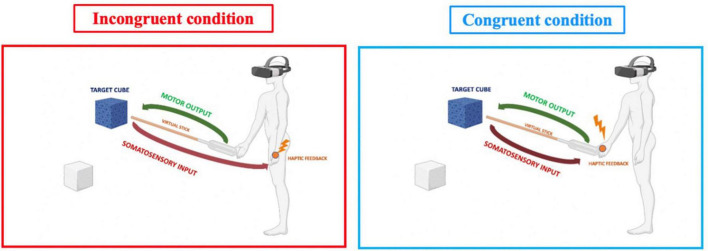
Experimental paradigm for congruent vs. incongruent condition.

The experimental design involved participants performing two virtual reality visual-motor-haptic tasks (congruent vs. incongruent) on different days. During each session, the temporal order judgment task was performed before and after the VR task. This design resulted in a 2-Way repeated measurement within participants, with the main factor of time (*pre vs. post)* and condition (*congruent vs. incongruent)*. The VR task had the same length and characteristics for both sessions, except for the spatial location of somatosensory feedback received during the task. Participants had to move a virtual cube toward a destination point using a virtual stick controlled by the right hand. Whenever the virtual stick touched the cube, haptic feedback was provided in two possible body parts (congruent vs. incongruent with the effector, depending on the experimental session). In the incongruent condition, the collision between the cube and the virtual stick caused a somatosensory input in the participant’s left hand, generating a sensorimotor conflict. In the case of the congruent condition, haptic feedback was provided to the right hand (which controlled the virtual stick), then congruent with the movement effector (Figure 1). During both conditions, the participant held both controllers but used only the right ones.

### Hardware and software

The VR equipment used for the experiment included a Meta Quest 2 HMD, with a resolution of 1920 × 1832 pixel per eye. The HMD was connected via Oculus Link to an Asus ROG Strix notebook, featuring an AMD Ryzen 9 5900HX CPU, 32 GB of RAM, a GeForce RTX 3080 GPU and a 17.3” screen with a resolution of 1920 × 1080 pixel. The virtual reality environment was developed with the Unity graphical Engine. The vibration system used for delivering tactile feedback was synchronized with the virtual environment. A coin-shaped vibrotactile actuator (3v) was used to provide the vibration and tapped into the controllers, in contact with the thenar of the right or left hand. We employed the thenar region of the hand to optimize the realism of feedback interaction with a tool, taking into account the hand’s position while grasping the tool. The position of the vibrotactile actuator was maintained the same across participants by marking it on the VR controller. The vibrotactile actuator was powered using an Arduino Uno board connected to the VR notebook, and synchronized with the virtual environment using the Uduino library.

### VR environment and task

**FIGURE 2:**
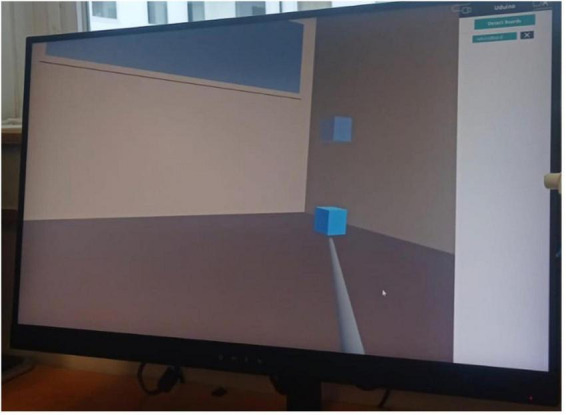
Example of trial during the VR task.

An empty room was used as the virtual space. When the participant wore the HMD and held both the left and the right controller. The experimenter instructed that only the right hand would be used during the task, and the left hand only kept the controller. Once the task started, a virtual stick appeared as the tool for interaction with the virtual environment. This was controlled by his/her right hand, but neither the left nor the right hand was shown in the virtual environment to prevent potential visual distractions/anchors during the task. Once the participant was familiarized with the virtual environment and the virtual stick, the experiment began with the first trial after pressing the controller button. At the beginning of each trial, a blue virtual cube appeared in the center of the virtual environment, while a target position cube appeared randomly in one of four locations in the virtual room (bottom-left, bottom-right, upper-left, upper-right). In both sessions, each participant received the same instruction: touch-and-move the blue cube using the virtual stick to match its position with that of the semi-transparent target cube. Every time the virtual stick collided with the blue cube, the participant received haptic feedback (to the right or the left hand, according to the experimental condition). Once the blue cube reached the semi-transparent target position cube, the trial ended, and a new blue cube appeared in the center of the virtual room (Figure 2). The task had a fixed duration of 10 min, and the experimental manipulation (i.e., the body part that the haptic feedback will stimulate) was masked until the beginning of the task.

### Temporal order judgment task

The temporal order judgment (TOJ) was programmed using Opensesame software connected to Arduino. Vibrotactile stimulation was provided to the left and right hand using 3v coin-shape vibrotactile actuator. The participant’s response was collected using two-button responses from the left and right thumb. During the task, the participant was seated in a comfortable position, with eyes open and gaze directed to the fixation cross in front of the screen. Participants performed 12 blocks of TOJ task before and 12 blocks after the VR task. In 6 blocks, participants had to indicate, pressing the left or right button, which hand received the first vibration. In the other 6 blocks, the participants indicated which hand received the second vibration. The order of blocks for “response to first” and “response to second” was counterbalanced across participants. Before a new block, the experimenter repeated the instructions for the task, as well as if it is required to respond to the first or respond to the second. Each block consisted of 16 trials, using 8 different SOA (stimulus onset asynchrony) (−600, −400, −250, −100, −70, −50,0.30, −15, + +15, + 30, + 70, + 100, + 250, + 400, + 600; negative values mean left first). The participants were instructed to respond as accurately and faster as possible. If the response was provided outside the range of 1000ms, the trial was considered invalid.

### Measurements

1. **Self-report VR experience**: four 6-point Likert items were employed to measure (1) the spatial and (2) temporal contiguity between interaction and tactile feedback, (3) body perception, and (4) the subjective unpleasantness of the overall experience. The range of responses goes from −3 (completely disagree) to 3 (completely agree).
2. **Performance during VR task:** the VR task’s performance was assessed by recording the number of trials completed within 10 min and the number of tactile interactions required for each trial for the congruent and incongruent conditions.
3. **Temporal order judgment:** tactile localization performance was evaluated through a Temporal order judgment (TOJ) task. Two measures were utilized for this task: the tactile sensitivity, known as the Just notable difference (JND), and the point of subjective equality (PSE). The JND represents the smallest detectable difference between two stimuli a person can perceive. In this specific case, the JND represented the temporal delay (in milliseconds) between two stimuli in which the participants correctly discriminated in 84% of the cases if the first (or the second) was provided to the left or to the right hand. The PSE represents the delay at which two different stimuli are perceived as equal by the observer. Specifically for the TOJ, the PSE should be placed around 0ms, and any difference reflects a bias toward one of the two hemispaces. These measures collectively quantified the sensitivity and bias in tactile localization performance when two rapid stimuli were presented subsequently to the right and left hand, as in the case of the temporal order judgment task (Heed and Azañón, 2014).

### Data analysis

The probabilities of order judgments within all conditions (Congruent Pre-VR, Congruent Post-VR, Incongruent Pre-VR, and Incongruent Post-VR) were modeled using a cumulative density function derived from a Gaussian distribution. The logistic regression model measured the point of subjective equality and just notable differences for each participant at each time (pre vs. post) for the two conditions (congruent vs. incongruent). PSE and JND data were submitted to a 2 × 2 repeated measurement ANOVA, with *time* (pre vs. post) and *condition* (congruent vs. incongruent) as within factors. In case of a significant interaction effect, planned contrast for each time-point (pre vs. post) was applied to compare congruent vs. incongruent conditions. Performances during the VR task (number of touches and trials) and questionnaire scores violated the normality assumption (Shapiro-wilk test > 0.05) and thus analyzed using the Wilcoxon *t*-test for non-parametric distribution, with the main factor of condition (congruent vs. incongruent). The significance level was set to 0.05, and the generalized eta square was used to report the effect size.

## Results

### Questionnaires

1. SPATIAL CONTIGUITY: No main effect of Condition on spatial contiguity was found (V = 37.5, *p* = 0.710).
2. TEMPORAL CONTIGUITY: No main effect of Condition on temporal contiguity was found (V = 10.5, *p* = 1).
3. BODY PERCEPTION: No main effect of Condition on body perception was found (V = 14.5, *p* = 0.660).
4. UNPLEASANTNESS OF EXPERIENCE: No main effect of Condition on body perception was found (V = 17, *p* = 0.203).

**FIGURE 3:**
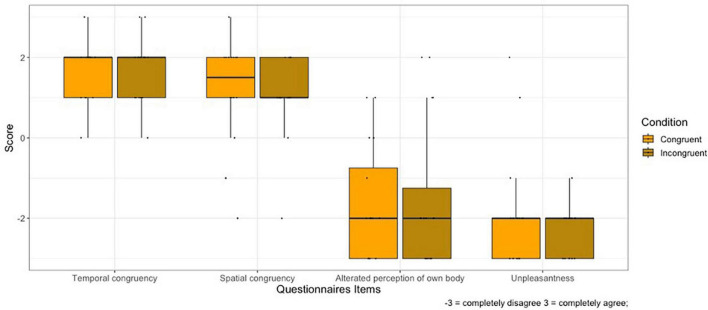
Rating of self-report questionnaires for the Congruent and Incongruent condition. Whiskers represent 1.5 IQR of the upper and lower quartiles.

Figure 3 shows the plot of self-reported questionnaires responses.

### Virtual reality task

**FIGURE 4:**
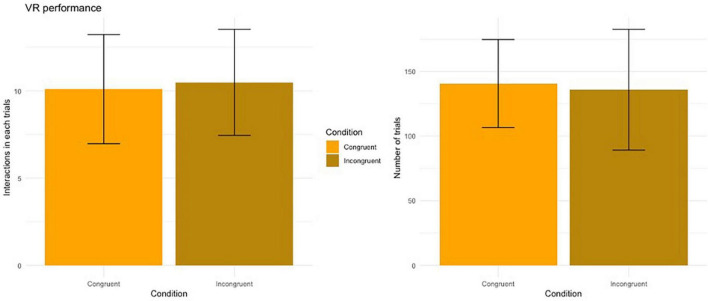
Performance during the VR task. Error bars represent the standard deviation.

*Number of touches in each trial*: No main effect of Condition on the number of touches performed to complete each trial was found (V = 77.5, *p* = 0.743). Participants touched the cube, on average, 10.3 times (SD = 3.02) during the congruent condition and 10.5 touches (SD = 2.90) for the incongruent condition (Figure 4, left panel).

*Number of trials completed*: No main effect of Condition on the number of trials completed during the VR task (V = 88.5, *p* = 0.913). Participants performed, on average, 140 trials (SD = 32.6) during the congruent condition and 116 trials (SD = 44.9) for the incongruent condition (Figure 4, right panel).

### Temporal order judgment

**FIGURE 5:**
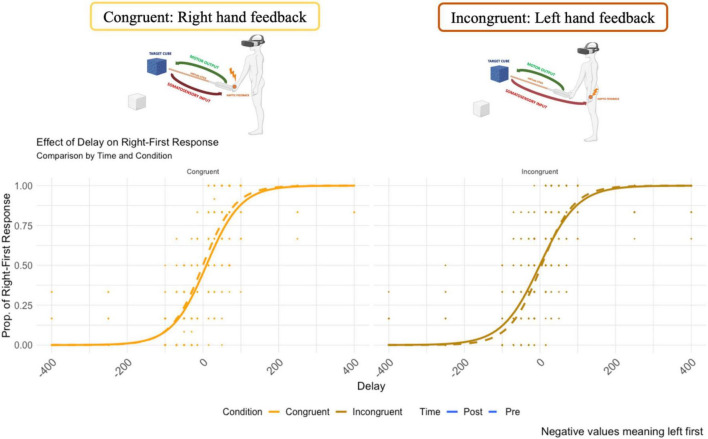
The psychometric curve of the responses for the Congruent and Incongruent condition (dash line represents the pre-VR assessment.

The psychometric function of the temporal order judgment is reported in Figure 5. The y-axis represents the probability of “right response first”. On the x-axis, the temporal delay between the administration of the two tactile stimuli (negative values mean left first).

### Point of subjective equality (PSE)

**FIGURE 6:**
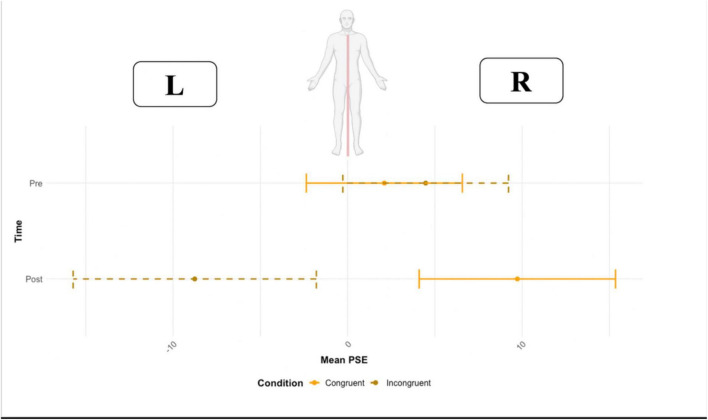
The point of subjective equality (PSE) before and after the Congruent and Incongruent conditions. Error bars represent the standard deviation.

No main effect of time (*F* = 0.52, df = 1,15, *p* = 0.821, ω2 = 0.004) or condition (F = 1.54, df = 1,15, *p* = 0.232, ω2 = 0.035) on the point of subjective equality was found. However, the two-way interaction time * condition was significant (*F* = 14.62, df = 1,15, *p* = 0.001, ω2 = 0.057). A pairwise t-test (Bonferroni corrected for multiple comparisons, p-value set to 0.025) was used to compare the PSE in the two conditions (congruent vs. incongruent) before and after the VR task. Before VR, no significant effect of condition was found (*p* = 0.689, congruent M = 2.10 ms, SD = 28.9 vs. incongruent M = 4.47, SD = 32.9), resulting in a comparable PSE before the VR task (close to zero). Crucially, after VR, the condition resulted in a significant effect (*p* = 0.022, congruent M = 9.72 ms, SD = 27.4 vs. incongruent M = −8.72 ms, SD = 34.2) (Figure 6). The PSE, after being exposed to the incongruent condition, was located toward the left side of the body (negative values represent left first), and to the right side of the body after the congruent condition).

### Just notable difference (JND)

**FIGURE 7:**
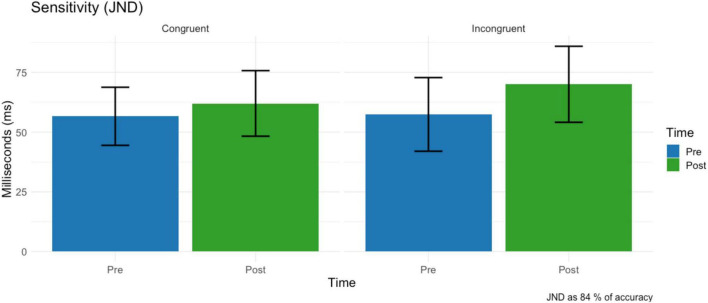
Just notable difference before and after the VR task for the Congruent (left) and Incongruent (right) conditions before and after the VR task. Error bars represent the standard deviation.

No main effect condition (*F* = 0.73, df = 1,15, *p* = 0.404, ω2 = 0.001) on just notable difference was found. A non-significant trend of time on JND (*F* = 4.33, df = 1,15, *p* = 0.054, ω2 = 0.006) revealed that tactile sensitivity decreased after the virtual reality task (M = 66.0 ms, SD = 56.5) compared to the baseline (M = 64.3 ms, SD = 56.5) (Figure 7). The two-way interaction time * condition was not significant (F = 1.31, df = 1,15, *p* = 0.269, ω2 = 0.001).

## Discussion

The study aimed to assess the presence of possible variations in tactile spatial localization processing performance arising from prolonged exposure to discrepancy between the movement effector (continuously providing proprioceptive information) and the corresponding body part receiving tactile feedback during an interaction. In two experimental sessions, participants performed the temporal order judgment task before and after a visuomotor task in virtual reality. During the VR task, participants interacted with their right hand to move a virtual cube in the space. The interaction with the cube could be characterized by tactile feedback provided in the same (right hand) or different (left hand) location regarding the body part that interacted with a virtual cube. Unlike prior research focusing on spatial hand positioning, our approach involved crossing the locations of tactile feedback during a motor interaction between both hands. This manipulation introduced a misalignment between the visual-motor-proprioceptive cues from body movement and the somatosensory feedback received. To explore potential after-effects in tactile localization, we compared the point of subjective equality (PSE) and just noticeable difference (JND) before (baseline) and after the incongruent (right-hand interaction, left-hand feedback) or congruent (right-hand interaction, right-hand feedback) condition of the VR-task.

As the main finding, the results revealed changes in the point of subjective equality (PSE) after exposure to a mismatched sensorimotor interaction, compared to the control condition. In a classic temporal order judgment, the PSE measures spatial bias toward a particular side of the body space (left vs. right body part), typically considering the midline as zero (Heed and Azañón, 2014). The PSE represents the stimulus intensity whereby two tactile stimuli (or, more generally, two stimuli) are perceived as arriving first on the left side of the space in 50% of the trials (Wada et al., 2004; Moseley et al., 2009). In other words, the PSE in a TOJ task represents where the point of max uncertainty between two stimuli is located in the space. In the baseline assessment (before the task), the Point of Subjective Equality (PSE) showed comparable values across the measurement (considering that the first recording was performed without any experimental manipulation). However, after the VR task, the PSE moved in opposite directions and depended on the body part stimulated by the somatosensory feedback during the motor interaction: the exposure to crossed tactile feedback (right-hand interaction with left-hand tactile feedback) resulted in a significant shift of the PSE toward the left side of the body. Conversely, in the congruent condition, there was a shift in the PSE toward the right side of the body. Sensitivity analysis revealed a marginal effect (*p* = 0.054) related to time, wherein the just noticeable difference (JND) increased, indicating a decline in performance after the task compared to the baseline. It’s crucial to emphasize that, in both conditions, the task consistently provided somatosensory feedback to one side of the body. Consequently, the temporal delay in which participants could clearly discriminate between left and right stimuli may have been compromised (albeit minimally) by unilateral somatosensory stimulation. Notably, the performance for the VR task remained consistent across congruent and incongruent conditions in terms of the number of trials completed. This suggests that incongruent conditions did not have a discernible impact on visuomotor performance. Furthermore, it is unlikely that the observed effect can be attributed to a different number of interactions, such as the number of stimulations received, between the two conditions.

The different directions of PSE found here might be taken to suggest that the visuo-motor task in VR, together with somatosensory feedback, has triggered some form of reorganization of the spatiotopic representation of the body. The drift in PSE might be interpreted as a “sensorimotor adaptation” of the body coordinates in space caused by a shift in the somatosensory feedback toward the left hand. Notably, in a classical TOJ assessment, the PSE is informative about bias toward one body’s hemispace. Indeed, the switch of the tactile feedback to the left hand during right-hand interaction might have caused a perceptual drift of the body midline toward the left body-hemispace, similar to what happens after prismatic adaptation. The prismatic adaptation (PA) involves the subject pointing toward a visual target using their hand while wearing prismatic goggles that unilaterally shift the visual field either to the right or left (Clarke and Crottaz-Herbette, 2016). The shift caused a deviant visual field toward one side of the space (left or right) while participants were required to point to one target in the space. After some adaptation trials, participants can realign the sensorimotor coordinates to compensate for the prismatic effect. Notably, after removing the prism-glasses, the aftereffect due to this visuomotor adaptation can be observed in pointing tasks, resulting from body midline (and visual stimulus motor localization) shifts toward the left side. Prismatic adaptation after-effect is currently applied to spatial neglect recovery to restore spatial attention from the left side of the space in neglect patients (Serino et al., 2006). A benefit from neglect symptoms has been reported after a single session of PA (Rode et al., 2001), and prismatic treatment demonstrated long-term effects lasting up to 6 months (Serino et al., 2007). Regarding the VR task used in this study, it can be considered a visuo-motor task, similar to what is performed for prismatic adaptation. Participants saw and moved the right hand (represented in the right body space) to interact with the cube, but the tactile feedback was consistently provided to the left body space, maintaining the temporal binding with the action performed. Remarkably, a brief duration of 5 to 7 min was found to be sufficient for enhancing visuo-spatial abilities in patients with neglect (Farnè et al., 2002), and our VR task lasted 10 min, resulting in a comparable amount of time. In our experiment, the switch of somatosensory feedback toward the left hand induced a realignment of the body midline as a form of visuo-motor tactile adaptation to the virtual interaction, indexed by the shift of the PSE toward the left hand. Another possible explanation considers the shift in PSE as the result of “disownership” for the right-hand representation, and the left-hand became dominant in tactile competition for short SOA trials. The lack of any tactile interaction on the right hand, but instead, the motor outcome bonded with the left tactile feedback, might have induced a loss of ownership of the right hand, together with realignment of the boundaries that define the right and left-hand space. However, the visual, proprioceptive, and motor signals were maintained congruent during the task and clearly visible to the participants, turning in a less plausible explanation for hand disownership in accounting for this effect. In a previous investigation, Folegatti et al. (2009) tested two conditions of visual-proprioceptive conflict on tactile perception: one involving the rubber hand illusion (i.e., an experimental manipulation leading to disownership of one’s own hand) and another one based on prismatic displacement, which, however, did not involve disownership of one’s hand. In both conditions, reaction times for tactile stimuli provided to the real participant’s hand were slowed down compared to the control condition (no visuo-proprioceptive conflict), even though the latter did not induce disownership of the own hand. That is, disownership is not a mandatory condition to find affected sensory processing for a specific body part. Specifically, in our study, the VR paradigm involved consistent and indiscernible proprioceptive and motor components during the task execution (Tuthill and Azim, 2018). That is, participants actively experienced sensorimotor contingencies between their actions and their consequences in the (virtual) external environment, resulting in a less plausible condition of disownership for their own bodies.

It is worth noting that our paradigm involved a sensorimotor task that is not fully comparable to the classic crossing-hand paradigms. While cross-the-hands did not involve a motor component, which is entirely passive, our paradigm involved a multisensory conflictual interaction between visual motor and somatosensory components. In the majority of the studies reported in the literature, the TOJ measurement is performed under crossed and uncrossed conditions, meaning that the impairment arises from an ‘online’ conflict (occurring during the task) in processing body-related spatial information (Yamamoto and Kitazawa, 2001; Shore et al., 2002; Schicke and Rôder, 2006; Moseley et al., 2009; Gallace et al., 2011; Sambo et al., 2013; Soto-Faraco and Azañón, 2013). Remarkably, in most cases, crossing hand posture caused a decrease in the JND and not the PSE, meaning a decrease in sensitivity to discriminate left-right stimulation as a consequence of a mismatch between somatotopic and spatiotopic reference systems. Instead, the outcome presented here concerned after-effects (following the VR task) in tactile processing, likely due to a realignment of visual-motor-proprioceptive and somatosensory information by sensorimotor interactions with one’s body. Notably, the impact on the PSE, as opposed to the JND, implies a reorganization of spatiotopic body coordinates. Indeed, the PSE is more indicative of the body midline and the left and right-side representation of the space around the body. This finding is crucial, as the after-effect resulting from a new sensorimotor binding in the output-input chain demonstrates the capability to reorganize the spatiotopic coordinates of the body.

While preliminary, practical implications can be inferred from the evidence presented in this study, particularly in clinical scenarios where somatosensory processing is impacted by a spatial bias toward one side of the body. This is the case of CRPS patients, as demonstrated by the study of Moseley and colleagues, where they prioritize stimuli coming from the non-affected side of the body (Moseley et al., 2009). However, the inversion of bias in tactile localization tasks when the hand are crossed suggesting the idea that CRPS symptoms may be attributed to a higher-order mechanism, extending beyond deficits to specific body areas and sensory modalities. Furthermore, the persistence of bias on the affected side of the body, even when the hands were crossed, implies a form of crystallization in the spatial representation of the body between the affected and unaffected sides. In this context, the possibility of modulating somatosensory processing by means of new (artificial) sensorimotor binding between affected and non-affected body parts could be useful in disrupting the crystallized representation between healthy and pathological body sides and modulating top-down mechanisms that support pathological conditions.

It is worth mentioning that the present study presents some limits that need to be addressed in the near future. In particular, the main limit regards the VR task, which was performed using a virtual stick instead of hand-free interactions, reducing the realism of the interaction. At the moment of investigation, virtual prototyping with hand-free interaction was not as accurate as we needed, introducing noise due to artifacts in movement and interaction (e.g., due to inverse cinematic). While a substantial body of evidence supports the embodiment and integration of tools in the body schema after the usage (Iriki et al., 1996; Maravita and Iriki, 2004; Miller et al., 2018), it remains plausible that this condition may have influenced the outcomes arising from spatial sensorimotor incongruence. Moreover, in the prototype used in this study, we preferred not to present virtual hands to avoid visual anchors during the VR manipulation to prevent attention mechanisms toward specific body parts; however, this could have potentially affected the realism of the interaction. Enhancements to the design of the sensorimotor experience, coupled with improvements in virtual prototyping features, are necessary for future studies. Also, simple vibrations could not correctly mimic the somatosensory stimuli involved in more real interactions, limiting the subjective experience of feedback incongruency. Indeed, no differences were reported in the subjective experience during the virtual reality. The perception of spatial and temporal contiguity between motor and tactile interaction was not significantly different for congruent vs. incongruent conditions. However, the scores for spatial contiguity were lower for the condition of crossed feedback. Similarly, there was no difference in altered body perception or tactile processing among our experimental conditions. Further investigations are then required since the use of our virtual reality paradigm (see also Girondini et al., submitted) has opened up new possibilities for innovative and advanced research paradigms in understanding the mechanisms of tactile localization and temporal perception under multisensory conditions of stimulus presentation.

## Conclusion

The primary focus of the study was to evaluate the existence of after-effects in somatosensory processing resulting from a sensorimotor realignment in spatial processing induced by visuomotor interactions and somatosensory feedback of one’s body movement. Using VR, it was possible to spatially shift the somatosensory feedback resulting from right-hand interactions toward the opposite hand. Compared to the baseline, participants showed a bias in left-right tactile localization measured using TOJ, whereas the body-midline shifted accordantly with the location of somatosensory feedback. These findings are informative regarding the possible reorganization of those body representation systems involved in tactile spatial localization, depending on the sensory features that characterize sensorimotor interactions.

## Data availability statement

The original contributions presented in the study are publicly available. This data can be found here: https://osf.io/gdx24/.

## Ethics statement

The studies involving humans were approved by the University of Milano-Bicocca, local ethical committee. The studies were conducted in accordance with the local legislation and institutional requirements. The participants provided their written informed consent to participate in this study.

## Author contributions

MG: Conceptualization, Data curation, Formal Analysis, Investigation, Methodology, Software, Writing−original draft, Writing−review and editing. MM: Software, Writing−review and editing. AG: Writing−review and editing.
